# Special

**Published:** 1888-11

**Authors:** 


### HALL'S JOURNAL OF HEALTH

TRUTH DEMANDS NO SACRIFICE; ERROR CAN MAKE NONE.

VOL. 35. NOVEMBER, 1888. No. 11.

### SPECIAL.

We call particular attention to the two advertisements in this number of our Journal, viz: THE FAMILY CYCLOPŒDIA OF USEFUL KNOWLEDGE and THE FARM, and HOUSEHOLD CYCLOPŒDIA.

These books are, indeed, all that is claimed for them, either one being regular $1.50 publications, though sold at the low price of $1.

Having completed special arrangement with the publisher, we are now in a position to make the following unprecedented offer to our patrons, which will stand open until January, 1889.

To each subscriber, remitting $1.25 by postal order or money direct to this office "Hall's Journal of Health" for one year, and the choice of either one of the advertised books. Or either of them, and our Journal for six months, on remitting $1. No discount from the above figures can be made, should the order call for both books, or any number of them, as our offer gives any single subscriber, the benefit of the lowest terms admissible, if ordered by the thousand. Sent to any address by mail, post paid. Observe that the remittance must be direct to this office.

So confident are we of the value of the above offer that any subscriber who feels that he has been deceived or imposed upon by it, on returning his *premium book*, and withdrawing his subscription, will have his money returned to him.

We ask each of our old subscribers to encourage this enterprise by sending us a new one.

